# Associations between gestational weight gain and weight development of the offspring: Differences depending on maternal pre-pregnancy BMI

**DOI:** 10.1007/s00404-024-07487-1

**Published:** 2024-04-13

**Authors:** Charlotte Barzen, Mandy Vogel, Wieland Kiess, Tanja Poulain

**Affiliations:** 1 LIFE Leipzig Research Center for Civilization Diseases, University of Leipzig, Philipp-Rosenthal-Strasse 27, 04103 Leipzig, Germany; 2 Department of Women and Child Health, Hospital for Children and Adolescents and Center for Paediatric Research (CPL), Leipzig University, Liebigstraße 20a, Haus 6, 04103 Leipzig, Germany

**Keywords:** LGA, Pregnancy, Maternal body mass index, Obesity, Birth weight

## Abstract

**Purpose:**

Obesity rates are rising, and the gestational weight gain (GWG) of most women does not comply with current guidelines. This study assesses the association of pre-pregnancy BMI (ppBMI) and GWG with the child’s weight development and investigates whether associations with GWG differ depending on ppBMI.

**Methods:**

Data were obtained from the cohort study LIFE Child (Germany), comprising 691 mother–child pairs. Children’s weight was followed until age five. Associations between maternal ppBMI, GWG, and children’s weight were evaluated using regression analyses.

**Results:**

The association between GWG and birth weight (BW) was significantly positive in normal and underweight (n/u) women (β_GWG_ = 0.05, *p* < 0.01, 95% confidence interval (CI) 0.03—0.07), but not in women with overweight or obesity (o/o) (β_GWG_ = 0.0002, *p* = 0.99, 95% CI −0.03 to 0.03). The risk of giving birth to an infant who was large for gestational age (LGA) increased with rising GWG in n/u women (OR = 1.6, *p* < 0.01, 95% CI 1.23—2.25). Women with o/o were at increased risk for a LGA baby regardless of GWG (OR = 3, *p* < 0.01, 95% CI 1.34—6.97). This trend persisted in the child’s weight development during the first 5 years of life.

**Conclusion:**

Women with o/o might increase their offspring’s risk for higher weight at birth and in early childhood. In n/u women, GWG might be the more influential factor. Women should strive for normal weight before conception and should be more attentive to GWG.

## What does this study add to the clinical work

**Table:** 

The results of our study show that gestational weight gain has a particularly large effect on children’s (birth) weight in women with normal weight. Women with overweight and obesity have a higher risk of bearing children with higher (birth) weight, but the risk does not increase with increasing gestational weight gain. In order to prevent high weight at birth and during early childhood, it is important to consider and monitor both maternal weight gain during pregnancy and maternal weight prior to pregnancy.	

## Introduction

Despite efforts to curb the obesity epidemic, overweight, or obesity (o/o) prevalence rates remain at a high level worldwide. In Germany, 67.1% of men and 53.0% of women are o/o, and obesity rates among young women (25–34 years), the age when most women consider conception, are rising [1, 2]*.* Studies indicate that women with o/o are at increased risk of bearing a child whose weight is large for gestational age (LGA) and who is o/o later in life [3]. Children of mothers with obesity are themselves at increased risk for long-term pediatric endocrine morbidity [4]. Moreover, women with o/o before conception suffer from an increased risk of adverse pregnancy outcomes such as gestational diabetes, labor induction, and neonatal hypoglycemia [5], irrespective of gestational weight gain (GWG) [6, 7].

Besides the effect of pre-pregnancy BMI (ppBMI), excessive GWG increases the risk for adverse pregnancy outcomes, LGA [8] and o/o in the offspring [9–11]. Similar to obesity prevalence, the number of women who experience excessive GWG is rising [12]. The GWG of two-thirds of women does not meet the current guidelines [13]. Especially pregnant women with o/o suffer from excessive GWG [12, 14]. Still, there is no international consensus on how much weight women should gain depending on ppBMI. In 2009, the Institute of Medicine (IOM, United States of America) published revised guidelines for optimal GWG based on ppBMI ranges for women with underweight (12.5–18 kg), normal weight (11.5–16 kg), overweight (7–11.5 kg), and obesity (5–9 kg) [15]. However, professionals are doubtful whether these recommendations truly capture optimal GWG and criticize them for insufficient detail [16–19].

How the association of GWG and (birth) weight (BW) differs depending on the ppBMI has yet to be fully clarified. Studies suggest that GWG has a strong effect in normal-weight women [8, 14, 20], but inconsistent associations are described for o/o women [6, 14, 21, 22].

Pregnancy might be a defining time for the offspring’s weight, with lifelong consequences. This prompted us to investigate the current distribution of GWG in Germany and whether women meet the current recommendations. We also assessed whether the described increased risks for higher BW and further weight development (0–5 years of age) due to increased maternal ppBMI and GWG could be confirmed in our study population. More specifically, this study investigates the not entirely clarified moderating effect of ppBMI on the association between GWG and BW and offspring’s later BMI. Given that studies have shown an association between socio-economic status (SES) and weight status, we also examined whether maternal SES might be associated with BW or subsequent weight development of the child [23].

## Methods

### Study design and setting

For the outlined project, we retrieved data collected within the LIFE Child study conducted at the Research Center for Civilization Diseases at Leipzig University, Germany. The LIFE Child study is a large population-based cohort study [24, 25], established in 2011, which examines factors (e.g., nutrition, physical activity) that contribute to the occurrence of civilization diseases such as obesity, diabetes, and cardiovascular diseases during infancy, childhood, and adolescence (0–21 years).

In order to explore how conditions during pregnancy impact the health of children, the LIFE Child study assesses pregnant women during their 24th and/or 36th week of gestation. Infants are examined at the age of 3, 6, and 12 months, and thereafter once per year until age 21. In general, parents and children who suffer from chromosomal or syndromic diseases are excluded from the LIFE child study.

Participants are mainly recruited via advertisement and word of mouth. All parents provided informed written consent before participation. This study was designed in accordance with the Declaration of Helsinki and the study program was approved by the local ethics committee (Reg. No. 477/19-ek).

### Study population and sampling procedure

Data from 804 mothers and their children, collected until 2020 (before the COVID-19 pandemic), were analyzed. From this dataset, 113 mother–child pairs were excluded due to conditions which might influence weight development during pregnancy (e.g., gestational diabetes) or weight development of the child (e.g., preterm birth). More specifically, we excluded twins and triplets, women and children with diabetes (Type I, Type II and gestational), and children who were born preterm (< 37 weeks of gestation; < 260 days) or post-term (> 42 weeks of gestation; > 293 days). Furthermore, outliers were removed (GWG > 50kg, *n* = 1, and weight loss during pregnancy, *n* = 2). The final dataset included 691 mother–child pairs.

In Germany, 10 preventive medical check-ups (“U-examinations”) screen children for developmental impairments or diseases. In this study, we assessed children’s BW (*n* = 691) and their BMI at nine time points documented in the preventive check-up booklet, namely, 3–10 days after birth (*n* = 452) and at 4–5 weeks (*n* = 688), 3–4 months (*n* = 654), 6–7 months (*n* = 573), 10–12 months (*n* = 458), 21–24 months (*n* = 323), 34–36 months (*n* = 238), 46–48 months (*n* = 169), and at 60–64 months (*n* = 92). The decrease in the number of available data with increasing child age can be explained by the decreasing (re-)participation of older children in the LIFE Child study.

### Measures

The data on maternal age, height, and weight were obtained from the maternity log, collected by the attending gynecologist at all prenatal examinations. PpBMI was calculated as weight before pregnancy (kg) divided by the square of height (m^2^). PpBMI was analyzed as a continuous variable or as a categorical variable (ppBMI category), with the two subgroups n/u weight (< 25 kg/m^2^) and o/o (≥ 25 kg/m^2^). The GWG period was defined as weight development from the last reported weight before pregnancy to the last reported weight during pregnancy, if measured less than 21 days before the delivery date.

The BW and BMI of the children documented at the time points of the “U-examinations” were used for the analyses, after transforming the weight and BMI measurements into age- and gender-adjusted standard deviation scores (SDS) according to the German growth standard (Kromeyer-Hauschild) [26]. At birth, we decided for BW and against BMI as length measurement at birth can be inaccurate. BW was additionally grouped into three categories: large for gestational age (LGA; BW-SDS ≥ 1.28), appropriate for gestational age (AGA; BW-SDS ≥  − 1.28 to 1.28), or small for gestational age (SGA; BW-SDS <  − 1.28).

SES was measured as a composite score combining information on parents’ education, professional position, and family income [27]. The score (range 3–21) can be used to categorize a family’s SES as high, medium, or low. Given the low percentage of low SES families in the present sample (1%), we combined low and middle SES and compared this group with the high SES group.

### Statistical analysis

All analyses were performed using R version 4.2.1. Data were described in terms of mean/SD (continuous measures) and numbers/percentages (categorical measures). Associations between the independent variables (age, ppBMI, GWG, and SES) and the dependent variables (weight [status] at birth and BMI-SDS at U-examinations) were assessed using univariate linear (for BW and BMI-SDS) and logistic (for weight status at birth) regression analyses. In all analyses, the effects of GWG were reported by five kg interval. Furthermore, all associations were checked for interactions between the mother’s ppBMI category (o/o versus n/u weight) and GWG. The ppBMI categories overweight and obesity as well as underweight and normal weight were combined as the effects did not differ between these categories. Models were checked for variance inflation using the generalized variance inflation factors (GVIF^(1/2*Df)) < 2). Given that the interactions were significant in most analyses, we reported associations between GWG and the dependent variables separately for each ppBMI category.

## Results

### Participants’ characteristics

**Fig. 1:**
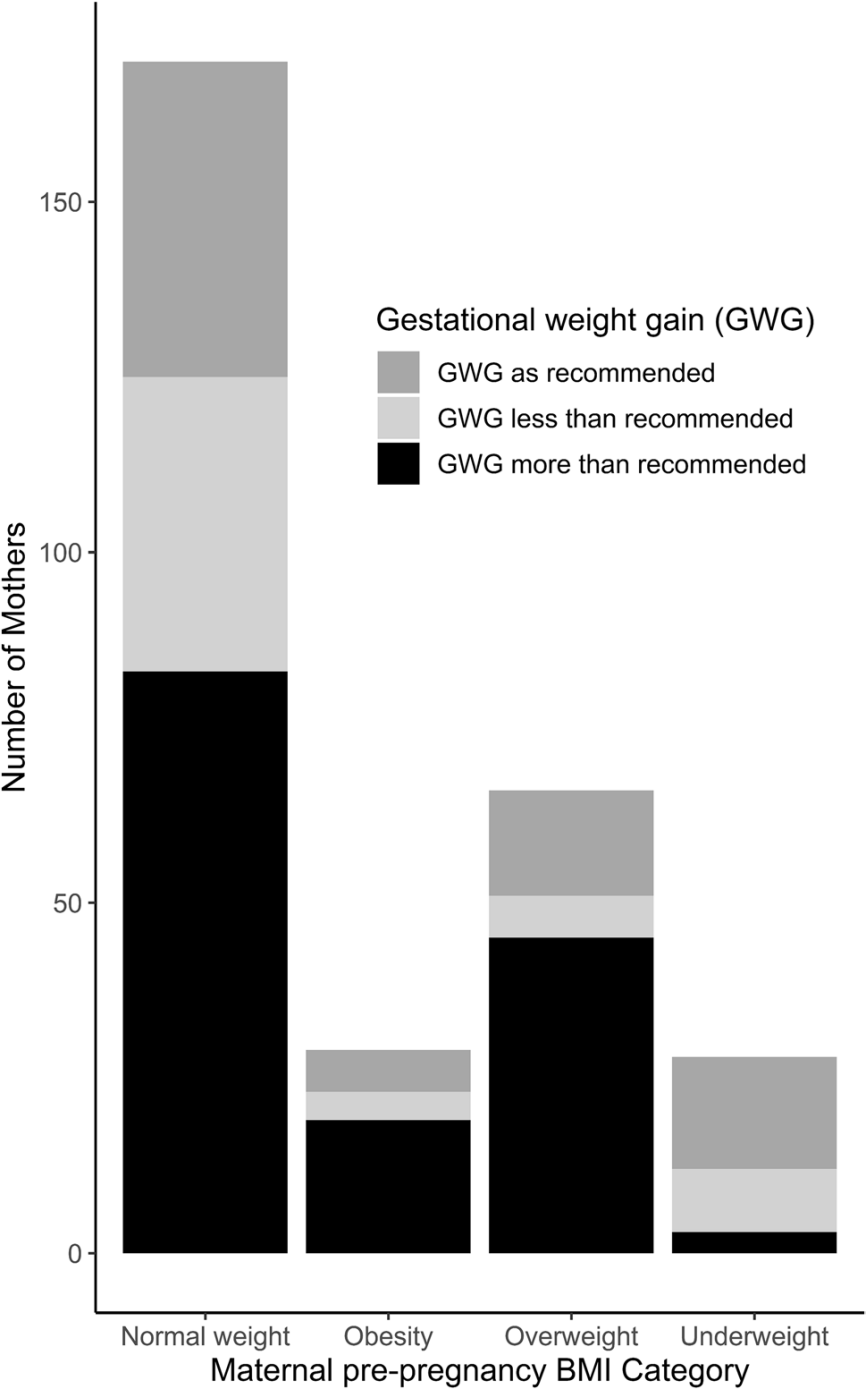
Women’s adherence to the IOM guidelines regarding their GWG, differentiated by their ppBMI category. *N* = 3 women with underweight, *n* = 83 with normal weight, *n* = 45 with overweight, and *n* = 19 with obesity exceeded the recommended limits (i.e., more than 18 kg, 16 kg, 11.5 kg, and 9 kg, respectively)

The total sample consisted of 691 mother–child pairs. Table 1 shows the demographic characteristics of the sample. The mean BW-SDS was 0.17. Five percent of the children were SGA, 82% were AGA, and 12% were LGA. The mean maternal ppBMI was 23.2 kg/m^2^. Most mothers (72%) were normal weight, 6% underweight, 16% overweight, and 6% obese. The GWG of women varied considerably from 0.4 to 34 kg, with a mean of 15kg. Figure 1 depicts women’s adherence to the IOM guidelines regarding their GWG, differentiated by their ppBMI category: most women did not meet the IOM recommendations [15] for GWG: 10% of underweight, 49% of normal weight, 68% of overweight, and 66% of obese women exceeded the recommended limits (i.e., more than 18 kg, 16 kg, 11.5 kg, and 9 kg, respectively).

**Table 1:** Cohort-specific description of sociodemographic and anthropometric data according to maternal ppBMI category (in Mean (SD) or number (%))

	[ALL] N = 691	Underweight N = 42	Normal weight N = 496	Overweight N** = 110**	Obese N = 43
Birth weight SDS	0.17 (0.94)	−0.10 (0.73)	0.16 (0.93)	0.33 (1.01)	0.18 (0.98)
Birth weight group:					
Appropriate for gestational age (AGA)	566 (81.91%)	38 (90.48%)	408 (82.26%)	87 (79.09%)	33 (76.74%)
Large for gestational age (LGA)	82 (11.87%)	1 (2.38%)	57 (11.49%)	17 (15.45%)	7 (16.28%)
Small for gestational age (SGA)	36 (5.21%)	2 (4.76%)	27 (5.44%)	4 (3.64%)	3 (6.98%)
Missing	7 (1.01%)	1(2.38%)	4 (0.81%)	2 (1.82%)	0
Sex child:					
Female	330 (47.8%)	19 (45.2%)	232 (46.8%)	57 (51.8%)	22 (51.2%)
Male	361 (52.2%)	23 (54.8%)	264 (53.2%)	53 (48.2%)	21 (48.8%)
Gestational age (weeks)	39.5 (1.11)	39.9 (1.07)	39.5 (1.12)	39.5 (1.00)	39.6 (1.22)
Gestational Weight Gain (kg)	15.2 (5.33)	14.2 (3.05)	15.5 (5.09)	15.5 (6.50)	11.3 (5.31)
Maternal age in years:					
< 24	24 (3.47%)	1 (2.38%)	17 (3.43%)	3 (2.73%)	3 (6.98%)
25–29	184 (26.63%)	12 (28.6%)	127 (25.60%)	34 (30.91%)	11 (25.58%)
30–39	448 (64.83%)	28 (66.7%)	328 (66.13%)	66 (60.0%)	26 (60.47%)
> 40	31 (4.49%)	1 (2.38%)	23 (4.64%)	6 (5.45%)	1 (2.33%)
Missing	4 (0.58%)	0	1 (0.2%)	1 (0.91%)	2 (4.65%)
Maternal socio-economic status (SES):					
High	199 (28.80%)	13 (30.95%)	152 (30.65%)	24 (21.82%)	10 (23.26%)
Low/medium	309 (44.72%)	17 (40.48%)	215 (43.35%)	54 (49.09%)	23 (53.49%)
Missing	183 (26.48%)	12 (28.57%)	129 (26.01%)	32 (29.09%)	10 (23.26%)

Regarding SES, 29% of women had a high SES and 45% had a low or medium SES. In 26% of cases, SES information was unavailable. The mothers who participated in our study were aged from 20 to 46 years, with a mean of 31 years.

### Associations between maternal age, SES, and BW/BMI of the children

Maternal age was significantly associated with children’s BW-SDS (β_age_ = 0.02, *p* = 0.02, 95% CI 0.00–0.04), but not with the risk of LGA (OR = 1, *p* = 0.5, 95% CI 0.97–1.07). The associations between maternal age and children’s BMI-SDS at the different preventive check-ups only reached statistical significance at the time point of U9, i.e., at the age of 5 years (β_age_ = 0.04, *p* = 0.03, 95% CI 0.00–0.07). There were no significant associations between maternal age and BMI-SDS at time points U2 to U8 (β_age_ between −0.02 and 0.01, all *p* > 0.08).

There was no significant association between SES and BW of children (β_SES_ = −0.07, *p* = 0.4, 95% CI −0.24 to 0.14) or the risk of LGA (OR = 1.2, *p* = 0.5, 95% CI 0.69–2.08). Similarly, there was no significant association between SES and children’s BMI-SDS at time points U2–U9 (β_SES_ between −0.08 and 0.27, all *p* > 0.12).

### Associations between GWG, ppBMI, and BW

In women with o/o, BW was significantly higher than in women with n/u (β_o/o=_  + 0.43, *p* < 0.01). Significant interactions between GWG and ppBMI showed that the strengths of associations between GWG and BW differed by maternal ppBMI category. In women with n/u, GWG showed a strong association with BW-SDS (β_GWG_ = 0.05, *p* < 0.01, 95% CI 0.03–0.07). In these women, the estimated mean BW-SDS was −0.14 for a GWG of 10 kg and 0.36 for a GWG of 20 kg. In comparison, in women with o/o, GWG showed no significant association with BW-SDS (β_GWG_ = 0.0002, *p* = 0.99, 95% CI −0.03 to 0.03) and the estimated mean BW-SDS was 0.29, independently of GWG.

**Fig. 2:**
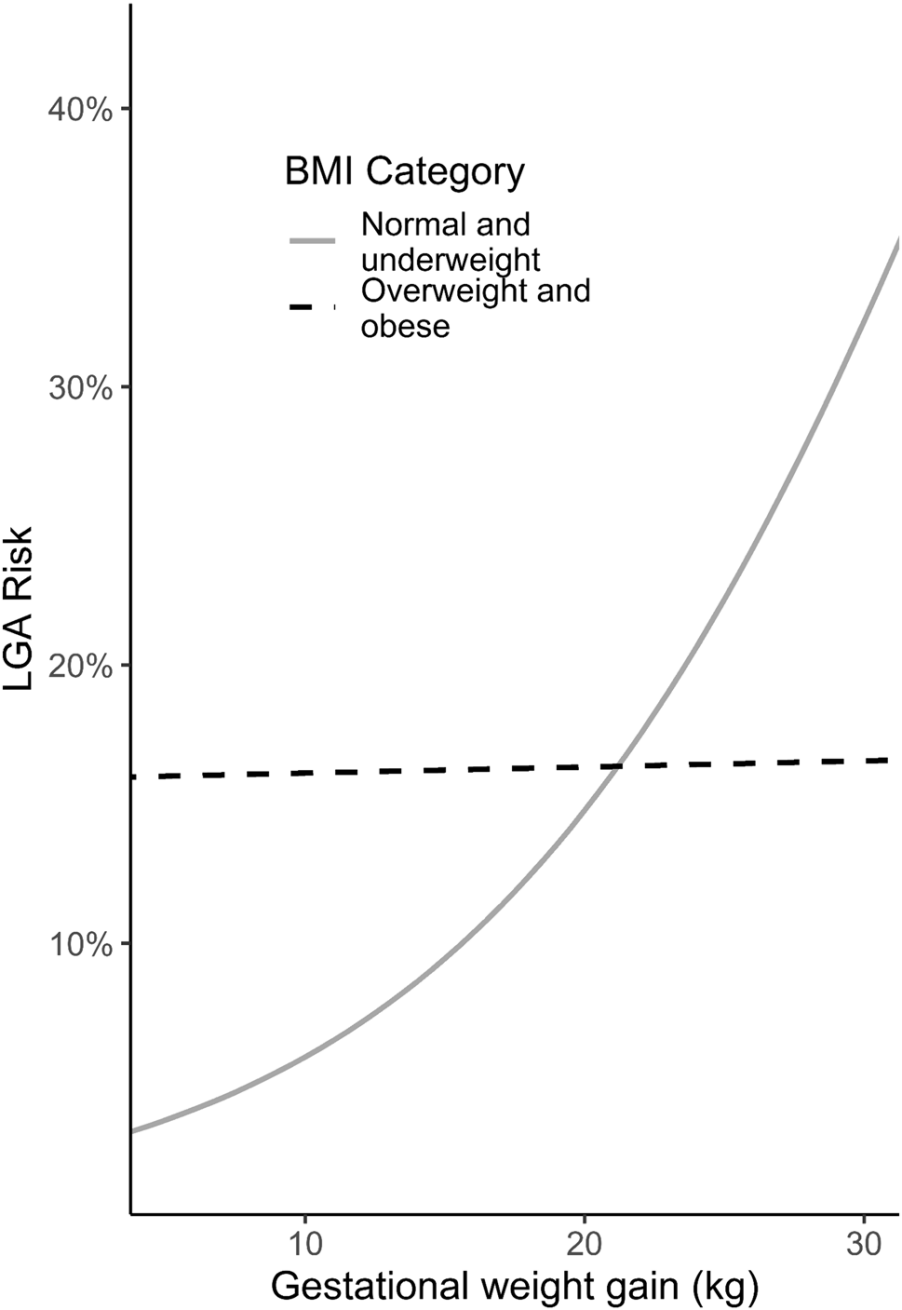
Associations between LGA and GWG in 5kg intervals depending on ppBMI category. The figure depicts an elevated risk for an LGA child among women with o/o compared to those with n/u. For women with n/u, the risk of having an LGA child significantly increases with increasing GWG

Logistic regressions revealed a higher risk of bearing a LGA child for women with o/o compared to women with n/u (OR = 3, *p* < 0.01, 95% CI 1.34–6.97). In women with n/u, the risk for LGA increased strongly with GWG (OR = 1.6, *p* < 0.01, 95% CI 1.23–2.25), as shown in Table 2 and Fig. 2. In women with n/u, the estimated risk of LGA was 6% for GWG of 10 kg, but 15% for GWG of 20 kg. In women with o/o, GWG was not significantly associated with the risk of LGA (OR = 1.01, *p* = 0.9, 95% CI 0.7–1.5). In these women, the estimated risk of LGA was 16%, irrespectively of GWG. The risk of LGA in mothers with n/u only approaches that of mothers with o/o if GWG is 20 kg or more (Table 2 and Fig. 2).

**Table 2:** Estimated LGA risk depending on GWG and ppBMI

Gestational weight gain (absolute)	Mothers with n/u	Mothers with o/o
+ 10kg	5.9%	16.1%
+ 20kg	14.7%	16.3%

### Associations between ppBMI, GWG, and children’s weight development (U2–U9)

**Fig. 3:**
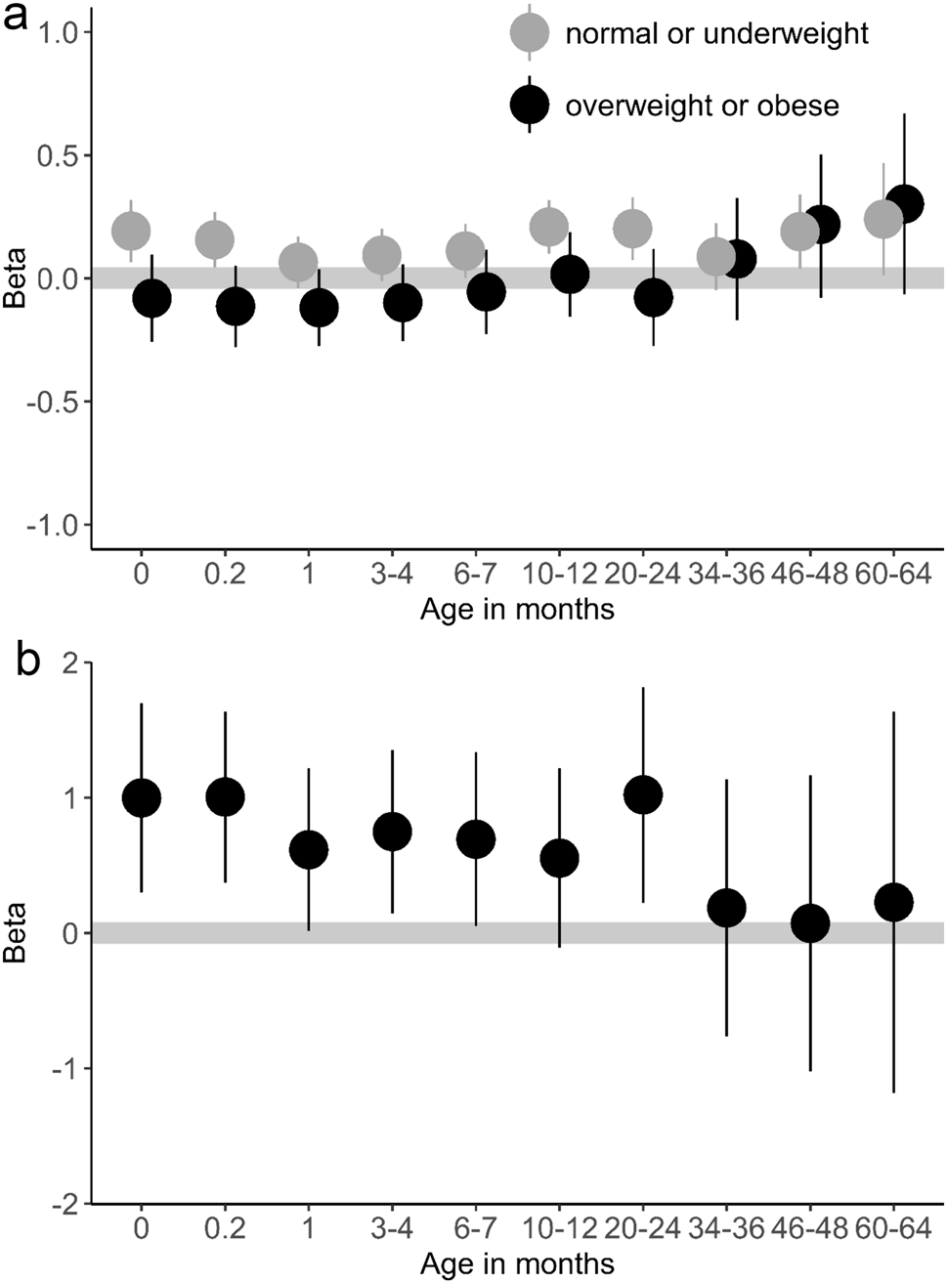
**a** Strength of association between GWG and child BMI-SDS at different time points (U1–U9) by ppBMI category: GWG in women with n/u before conception showed a positive correlation with the BMI-SDS of their children, with a nearly constant effect size. In contrast, in the group of mothers with o/o, GWG was not significantly associated with their children’s weight at any time point. **b** Strength of association between maternal o/o and child BMI-SDS: children of mothers with o/o had a higher BMI-SDS compared to children of n/u mothers

As with BW, GWG in women with n/u before conception was positively associated with the BMI-SDS of the children at 1 week to 5 years of age, with an almost constant effect size (β_GWG_ between 0.06 and 0.2, Fig. 3a). Statistical significance was reached at the time points of U2, U6, U7 (each *p* < 0.01), U8 (*p* = 0.01), U5 (*p* = 0.05), and U9 (*p* = 0.04) (Table 3). On the contrary, in mothers with o/o, GWG was at no time point significantly associated with children’s BMI (Fig. 3a and Table 3). However, children of mothers with o/o had a generally higher BMI-SDS than children of mothers with n/u (β_GWG_ between 0.07 and 1.0) (Fig. 3b).

**Table 3:** Association between GWG in 5kg intervals and BMI-SDS of children depending on maternal ppBMI category

Time point	Age (in months)	Mothers with n/u			Mothers with o/o		
		β_GWG_	p	95% CI	β_GWG_	p	95% CI:
U2	0.2	0.2	< 0.01	0.04–0.27	−0.11	0.2	−0.28–0.05
U3	1	0.06	0.22	−0.04 to 0.17	−0.12	0.1	−0.28—0.04
U4	3–4	0.09	0.08	−0.01 to 0.20	−0.10	0.2	−0.25—0.06
U5	6–7	0.11	0.05	0.00–0.22	−0.06	0.5	−0.23–0.12
U6	10–12	0.2	< 0.01	0.18–0.32	0.02	0.9	−0.16—0.12
U7	20–24	0.2	< 0.01	0.07–0.33	−0.08	0.4	−0.27–0.12
U7a	34–36	0.09	0.2	−0.05 to 0.22	0.08	0.5	−0.17–0.33
U8	46–48	0.2	0.01	0.04–0.34	0.22	0.2	−0.08—0.52
U9	60–64	0.2	0.04	0.01–0.47	0.30	0.1	−0.07–0.67

## Discussion

Our findings show that women with o/o carry an increased risk of giving birth to infants with higher BW compared to women with n/u [28, 29], regardless of GWG. Interestingly, our results suggest that in n/u mothers, GWG increases the risk of having a child with higher BW. These associations remain consistent in the first 5 years of age.

Despite initial expectations, no significant association was found between children’s (birth) weight and SES. This could potentially be explained by the fact that only 1.6% of women in the sample had a low SES.

### Associations between GWG, ppBMI, and children’s (birth) weight

Previous studies found that GWG was positively associated with infants’ BW [8, 28, 30] and with a higher risk of o/o throughout childhood [9–11]. Furthermore, higher maternal BMI before pregnancy was associated with higher child weight at birth and in later development [9, 31].

In the current literature, there is no consensus on how the maternal BMI level might modify the association between GWG and BW [6, 14, 21, 22]. In line with previous findings [8, 20], we found that GWG has a stronger effect on BW in n/u mothers than in mothers with o/o. Zhao et al. suggested that in normal-weight mothers, GWG could be an independent predictor for adverse BW [29]. In women with o/o, different associations between GWG and BW were described [6, 14, 21, 22].

Concerning the further weight development of children after birth, prior studies observed significant associations between excessive GWG and children’s BMI-SDS only in normal-weight mothers [31], or only small additional effects of excessive GWG in mothers who were already o/o [9].

### Biological mechanisms behind the association between excessive GWG, ppBMI, and (birth) weight

An explanation for why excessive GWG and maternal ppBMI are associated with children’s (birth) weight is provided by the development overnutrition hypothesis. Obesity, insulin resistance, and excessive GWG cause high glucose and triglyceride levels in pregnant women, which are transferred transplacentally, inducing higher levels of blood sugar and nutrients in the fetus [10, 32]. In response, the fetal pancreas starts to produce greater amounts of insulin, triggering fetal growth [29, 33–36].

Excessive GWG and ppBMI, as described before, cause higher maternal and fetal glucose levels, which may shape the fetal metabolism and organ and tissue structure through epigenetic mechanisms in utero [4]. This fetal programming could increase susceptibility to obesity throughout the offspring’s lifespan [37]. These explanations could also explain our findings on the association between ppBMI, GWG, and weight of children up to 5 years of age. Likewise, Arroyo-Jousse et al. indicate that maternal overnutrition and obesity could lead to higher expression of the hormones leptin and adiponectin in maternal and fetal adipose tissue and could modulate the placental function and fetal physiology, which may lead to obesity in later life [38].

One possible reason why the effect of GWG is particularly strong in women with n/u is that, before pregnancy, women with n/u usually have a healthier insulin and glucose metabolism than women with o/o. In n/u mothers, high GWG can initiate an adverse metabolic situation, whereas in mothers with o/o, the metabolic condition might already be adverse at the beginning of pregnancy. Our study reveals that an n/u mother only approaches the same risk of LGA as women with o/o if she gains more than 20 kg during pregnancy. This might indicate that a GWG of 20 kg leads to a similar metabolic situation as being overweight or obese.

### Strengths and limitations

The strengths of our study are the large sample size, the consideration of the effect of ppBMI category on the association between GWG and BW, and the investigation of weight development until age five. Nevertheless, there are several limitations. There is a bias in our study population, since mother–child pairs with lower SES were under-represented. Most participants lived in a large city. In addition, the exclusion of women with (gestational) diabetes as well as children born pre- or post-term created a supernormal collective. Moreover, not all confounding factors that might influence the association between GWG or ppBMI and children’s weight development were considered, e.g., maternal energy intake, smoking, alcohol consumption, movement, parity, birth order, or paternal BMI [39, 40]. Also, we did not distinguish during which period of pregnancy the weight was gained [8].

## Conclusion

The current study demonstrated that women with overweight or obesity are at increased risk for bearing children with higher (birth) weight, regardless of their gestational weight gain. In contrast, in women with normal weight, the risk for bearing children with higher (birth) weight increases with increasing gestational weight gain. Regarding public health implications, our study recommends two important endpoints of modifiable risk factors to consider: Health professionals should educate their patients in pre-conceptional care about the importance of achieving normal weight before conception and regulating their weight gain during pregnancy. More detailed recommendations for optimal gestational weight gain are needed.

## Abbreviations

GWG: Gestational weight gain
ppBMI: Pre-pregnancy body mass index
LGA: Large for gestational age
SGA: Small for gestational age
AGA: Appropriate for gestational age
n/u: Normal and underweight
o/o: Overweight and obesity
BW: Birth weight

## Declarations

### Availability of data and materials

The datasets generated and/or analyzed during the current study are not publicly available due to ethical restrictions. The LIFE Child study is a study collecting potentially sensitive information. Publishing datasets is not covered by the informed consent provided by the study participants. Furthermore, the data protection concept of LIFE requests that all (external as well as internal) researchers interested in accessing data sign a project agreement. Researchers who are interested in accessing and analyzing data collected in the LIFE Child study may contact the data use and access committee (forschungsdaten@medizin.uni-leipzig.de).
